# Alternative vaccine administration by powder injection: Needle-free dermal delivery of the glycoconjugate meningococcal group Y vaccine

**DOI:** 10.1371/journal.pone.0183427

**Published:** 2017-08-24

**Authors:** Nikolas T. Weissmueller, Leanne Marsay, Heiko A. Schiffter, Robert C. Carlisle, Christine S. Rollier, Robert K. Prud’homme, Andrew J. Pollard

**Affiliations:** 1 Department of Paediatrics, Oxford Vaccine Group, University of Oxford and the NIHR Oxford Biomedical Research Institute, Oxford, Oxfordshire, United Kingdom; 2 Department of Biological and Chemical Engineering, Princeton University, Princeton, New Jersey, United States of America; 3 Institute of Biomedical Engineering, Biomedical Ultrasonics, Biotherapy & Biopharmaceuticals Laboratory (BUBBL), Oxford, Oxfordshire, United Kingdom; Instituto Butantan, BRAZIL

## Abstract

Powder-injectors use gas propulsion to deposit lyophilised drug or vaccine particles in the epidermal and sub epidermal layers of the skin. We prepared dry-powder (T_g_ = 45.2 ± 0.5°C) microparticles (58.1 μm) of a MenY-CRM_197_ glyconjugate vaccine (0.5% wt.) for intradermal needle-free powder injection (NFPI). SFD used ultrasound atomisation of the liquid vaccine-containing excipient feed, followed by lyophilisation above the glass transition temperature (T_g_’ = − 29.9 ± 0.3°C). This resulted in robust particles (density~ 0.53 ±0.09 g/cm^3^) with a narrow volume size distribution (mean diameter 58.1 μm, and span = 1.2), and an impact parameter (ρvr ~ 11.5 kg/m·s) sufficient to breach the *Stratum corneum* (*sc*). The trehalose, manitol, dextran (10 kDa), dextran (150 kDa) formulation, or TMDD (3:3:3:1), protected the MenY-CRM_197_ glyconjugate during SFD with minimal loss, no detectable chemical degradation or physical aggregation. In a capsular group Y *Neisseria meningitidis* serum bactericidal assay (SBA) with human serum complement, the needle free vaccine, which contained no alum adjuvant, induced functional protective antibody responses *in vivo* of similar magnitude to the conventional vaccine injected by hypodermic needle and syringe and containing alum adjuvant. These results demonstrate that needle-free vaccination is both technically and immunologically valid, and could be considered for vaccines in development.

## Introduction

Most of the 800 million vaccine injections performed each year are administered by intramuscular needle and syringe injection [1]. There are a number of disadvantages associated with conventional needle administration, including the risk of transmission of blood-borne viruses (such as HIV), the need for large-scale disposal of needles [2–4]. Glycoconjugate vaccines comprise one or more polysaccharides of bacterial origin that are covalently linked to an immunogenic carrier protein [5]. They are thymus dependent vaccines, due to the protein carrier which recruits T cells to enhance the B cell response in germinal centres, and have substantially reduced the disease burden associated with polysaccharide-encapsulated bacteria, such as *Haemophilus influenza* type b, *Streptococcus pneumoniae* and *Neisseria menigitidis* [6–8]. Meningococcal meningitis and septicaemia, caused by *Neisseria meningitidis* (Men) infection, involves invasion of the blood and meninges of the brain and spinal cord and is accompanied by devastating morbidity and mortality. The largest disease burden historically fell on sub-Saharan Africa, also known as the meningitis belt, where a large-scale program is underway to protect the population from further outbreaks [9]. Of the pre-existing vaccines, none have a needle-free alternative.

A plethora of vaccine delivery approaches achieve permeation or penetration of the *Stratum corneum* (*sc)* barrier. These include chemical permeability enhancers, ultrasound, electroporation, micro-needles, liquid-jet injection, and *sc* removal by tape-stripping among others [10–13]. The advantages and disadvantages of these methods have been previously summarized. While no single needle-free technology is likely to emerge as the most suitable choice for all clinical settings, the respective advantages and limitations of each technology will differentiate their suitability [12]. One advantage of powder injection is the use of well-established and readily scalable pharmaceutical formulation processes such as lyophilisation [14, 15].

Needle-free powder injection (NFPI) is a delivery modality that deposits vaccine or drug particles into the epidermal and sub epidermal layers of the skin [16, 17]. Ballistic injectors expose individual powder particles to high acceleration and deceleration forces, as experienced upon device actuation and particle impact on the skin [18]. Powder injector devices accelerate vaccine particles in the stream of expanding helium gas (at 30–60 bar pressure), and achieve velocities of up to 1050 m/s [19]. According to Kendall et al, the impact parameter (ρrv) required for particles to breach the *sc* ranges from 7–12 (kg/m·s) and is a function of particle density (ρ), radius (r) and velocity (v).[18] Generally, particles less than 100 μm in diameter have been reported as pain-free, while particles smaller than 20 μm were unable to penetrate into the epidermis [20]. If the overall amount of powder per injection is kept below 1–2 mg, bleeding can be almost entirely avoided [16].

The epidermis is difficult to target, but has several favourable immunogenic properties. Phenotypically immature Langerhans cells (LCs) line the epidermis in a network of cytoplasmic processes, which cover about 25% of the epidermal area. Upon contact with an antigen, LCs endocytose the antigen. Subsequently, LCs detach from their cytoplasmic network, and prompted by inflammatory cytokine cues of the surrounding keratinocytes, LCs migrate into the lymphatic system where they mature to antigen-presenting cells (APCs), before interacting with naïve T cells in the lymph nodes [21]. Interconnected components of the cutaneous immune response extend from the site of pathogen entry, to tissue homing antigen specific cell mediated immunity, to systemic immunity of the entire organism and confer an unique immunological potency to the skin, which renders it an especially appealing target for alternate immunization procedures such as needle-free powder injection that may potentiate and modulate the immune response [22, 23]

Since 2003, most investigational dry-powder formulations of viral and bacterial protein antigens for application in needle-free ballistic injection were manufactured by spray-freeze drying (SFD) [24]. Briefly, SFD atomizes a liquid formulation that conventionally contains an active pharmaceutical ingredient and various excipients into a cryogenic liquid prior to the removal of water from the frozen droplets by ice sublimation at reduced temperatures and pressures [25]. Relative to alternative particle manufacture methods, such as spray-drying or compaction and grinding, SFD readily produces particles with a larger diameter and a more narrow size distribution respectively [26, 27]. However, many small ice crystals form within single particles due to fast freezing rates, which upon sublimation results in a highly porous construct that mechanically is not suitable for ballistic injection [28–30]. The addition of a high solute content in the liquid feed, the addition of polymers such as dextran, or a temperature adjustment to the lyophilisation procedure may be used to reduce particle porosity and increase structural robustness of SFD powders [15, 27, 31].

Ballistic injector formulations balance the physicochemical integrity of the active agent with the mechanical robustness of particles to breech the *sc* [17, 32–36]. A formulation developed specifically for use with needle-free particle injectors, tested in a successful phase I clinical study with an influenza vaccine, comprised trehalose, mannitol, and dextran (10kDa) at 35% by mass and produced Fluvirin^®^-loaded particles with a 39 μm diameter and a tap density of 0.72 g/cm^3^, yielding a 10.5 kg/ms impact parameter at the devices’ estimated exit velocity of 750 m/s [37, 38]. Later work optimized the SFD process parameters for the needle-free injection of glucagon-loaded particles, and added a higher MW dextran 150 kDa to the TMD formulation for increased mechanical robustness. The 35% by mass trehalose-mannitol-dextran_10kDa_-dextran_150kDa_ (TMDD) excipient matrix achieves the required physical characteristics and enables ballistic injection [27, 31].

Here we report the development of a needle-free TMDD formulation of the meningococcal serogroup Y glycoconjgate MenY-CRM_197_ with suitable physical characteristics for intradermal powder injection using spray-freeze drying. The chemical integrity of MenY-CRM_197_ after SFD was assessed using SDS-PAGE, and its physical integrity by asymmetric flow field-flow fractionation (AF4). An *in vivo* study was conducted and the immune response generated was assayed by ELISA and serum bactericidal assay (SBA). The efficacy of the needle-free formulation was compared to the current clinical standard, MenY-CRM_197_ (plus Alum-adjuvant) delivered by intra-muscular injection. This is the first account of needle-free inoculation by dry-powder injection with a complex glycoconjugate vaccine.

## Materials and methods

### Manufacture of spray-freeze-dried MenY-CRM_197_ powders

The investigational MenY-CRM_197_ vaccine had a polysaccharide-to-protein ratio of 0.625 by mass, contained 3mg/mL of the meningococcal serogroup Y polysaccharide (Men Y), 4.8 mg/mL *Cornybacterium diphtheriae* CRM_197_ protein, and was suspended in 10% (w/v) sucrose in 10 mM K_2_HPO_4_ buffer pH 7.2 (provided by Novartis Vaccines and Diagnostics, Siena, Italy). For comparison, the commercial vaccine Menveo^®^ (Novartis Vaccines) contains 10 μg Men A polysaccharide and 5 μg of the Men C, Men W and Men Y polysaccharides conjugated to a total of 32 – 64 μg CRM_197_, which corresponds to a polysaccharide-to-protein ratio of 0.39 – 0.78 by mass.

**Fig 1:**
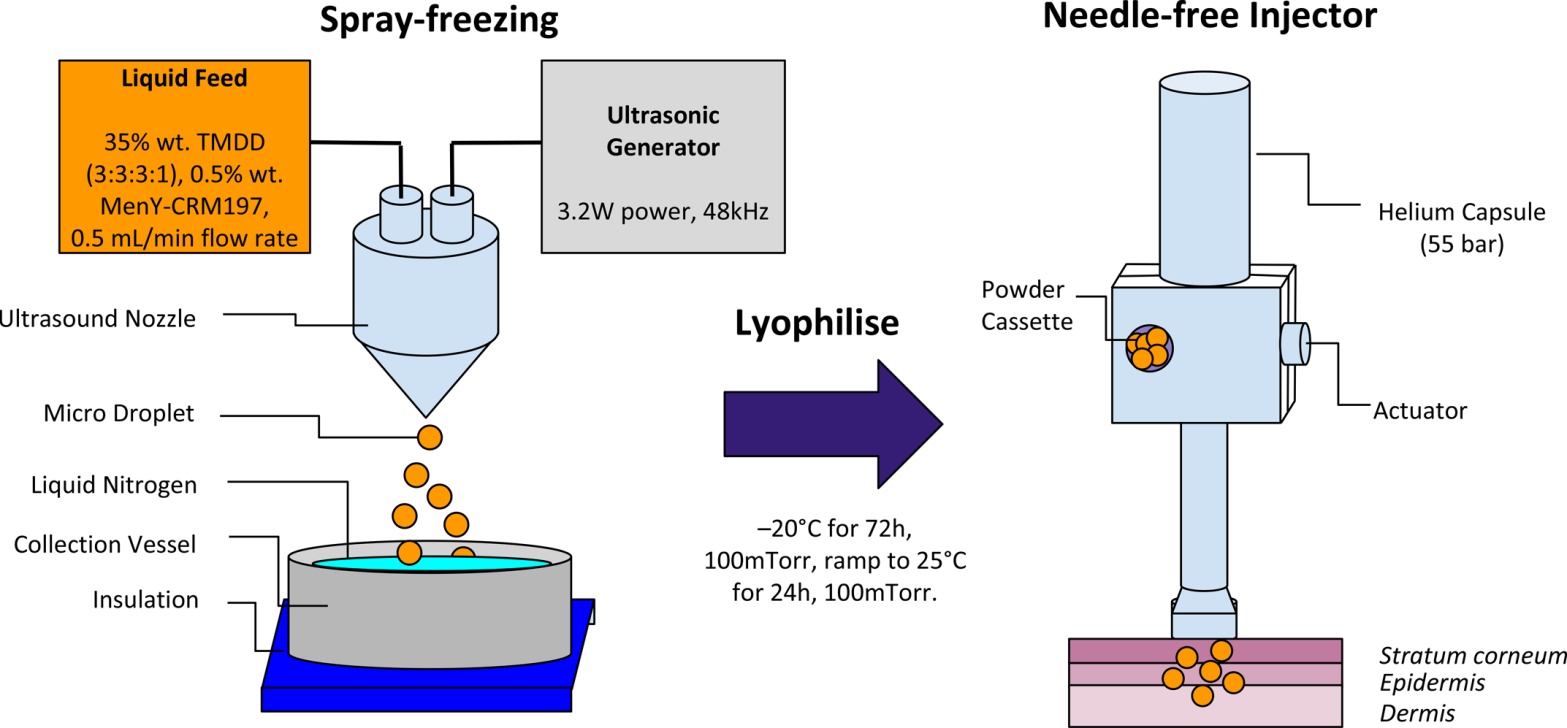
Spray freeze dry process for MenY-CRM_197_ powders. The spray freeze drying set-up used a 48 kHz ultrasound nozzle to atomise the 35% wt. TMDD (3:3:3:1) excipient solution containing 0.5% wt. MenY-CRM_197_, at 3.2W power and a 0.5mL/min flow rate. Particles were shock-frozen into liquid nitrogen and transferred into a pre-equilibrated LyoStar I and lyophilized. The product was filled into injector device powder cassettes, and stored at -80°C until use.

Dry-powder formulations of MenY-CRM_197_ for application in needle-free injection were prepared by a process previously described by Johnson et al. ([39]) and Costantino et al. [28, 29]. Formulations of trehalose, mannitol, dextran (10 kDa), dextran (150 kDa) (3:3:3:1) 35% (w/w) in 10 mM K_2_HPO_4_ buffer pH 7.2; contained 0.5% wt. MenY-CRM_197_ (mass of MenY-CRM_197_ to mass of excipients). Stainless steel collection vessels were fitted with precision fine wire thermocouples (Omega Engineering, LTD). Formulations were sprayed into collection vessels filled with liquid nitrogen (Fig 1), using a 48 kHz ultrasound nozzle (Sono-Tek, Milton, NY, USA) at 3.2 W (Broadband Ultrasounic generator, Sono-Tek, NY, USA) and a flow rate of 0.5 mL/min set by a Minipuls3 peristaltic pump (Gilson, Villers Le Bel, France). Once LN_2_ had boiled off, the collection vessels were sealed with a Gore Lyoguard lid (W.L. Gore & Associates, Newark, USA). The closed collection vessels were transferred onto pre-cooled shelves (– 40°C) and lyophilized in a FTS Systems LyoStar I (SP Industries, Warmister, PA, USA) at 100mTorr and a primary drying temperature of –20°C for 72 h. Secondary drying was conducted at 25°C for 24 h. All SFD formulations were lyophilized above their respective glass transition temperatures Tg’ to obtain less porous and more robust particles via viscous flow compaction of the amorphous glass [15]. The powders were transferred from the stainless steel collection vessels into freeze drying vials in a low humidity glove box at RT, sealed, crimped, and frozen to– 80°C for storage.

### Protein concentration determination by BCA assay

BCA was conducted according to supplier’s instructions (Bicinchoninic Assay Kit—QuantiProTM BCA Assay Kit, Sigma Aldrich, UK). Briefly, BCA working reagent was prepared from of a 1:1 dilution of solution QA with QB, and activated with copper (II) sulfate reagent QC in a ratio of 1:50 to QA-QB. CRM_197_ containing samples were added to the working reagent in a 1:1 ratio per well on an optically clear 96-well plate (Corning, UK). CRM_197_ concentration was determined relative to a BSA standard curve with concentrations 0, 0.5, 5, 10, 20, 30 μg/mL made in identical buffer to the samples. The plate was incubated at 37°C for 2h and absorbance was measured on a FLUOstar Omega (BMG Labtech LTD, Aylesbury, UK) plate reader at λ = 562nm. Concentrations were adjusted to blank and related to the BSA concentration curve.

### Turbidity measurements

Solution turbidity of ultrasound-atomized excipient solutions containing 2 mg/mL MenY-CRM_197_ was measured on a Varian Cary 50 Bio UV-Visible Spectrophotometer (Varian, Yarton, UK) with a quartz precision cell (Hellma, GER) of 10 mm light path at λ = 500 nm at RT. Untreated excipient-MenY-CRM_197_ solutions served as control. Turbidity is expressed as the absorbance value at λ = 500 nm.

### Determination of Tg and Tg’ for needle-free formulations

Differential scanning calorimetry (DSC) was used to determine the glass transition temperature (T_g_) of the dry-powder after SFD. For T_g_ determination, approximately 5 mg of SFD-powder was weighed, and sealed into the hermetic Tzero aluminium pans (TA Instruments, T111208) and the corresponding aluminium lid (TA Instruments, T1111019). The protocol equilibrated the sample at ‒10°C and held it isothermal for 20 min. The temperature was ramped to 90°C at 10°C/min. Analysis of the thermal signals was performed with TA Universal Analysis 2000 Software (Version 4.7A). The heat capacity thermogram (J/g°C) was plotted and used for analysis. The temperature at the inflection point of the endothermic shift in heat capacity was reported as the glass transition temperature. For T_g_’ determination, 20 μL of excipient solution with either 2 mg/mL CRM_197_, or without, was pipetted into hermetic Tzero aluminium pans (TA Instruments, T111208) and hermetically sealed with the corresponding aluminium lid (TA Instruments, T1111019). Samples were analysed on a Q2000 DSC, and computer operated with Thermal Advantage Software interface Version 2.8. Samples were equilibrated at ‒ 60°C and held isothermal for 20 min. The temperature was then modulated with ± 0.5°C every 100 seconds. After 5min isothermal, the temperature modulation had stabilised. The temperature was ramped to 0°C at 1°C/min.

### Helium-pycnometry and tapped density measurements

The density of powders (ρ He) was measured with a helium pycnometer (Pycnomatic ATC, ThermoFisher, Loughborough, UK). The measurement chamber was fitted with a small volume vessel unit (6.95255 cm^3^). The sample chamber volume was further reduced by 3.28743cm^3^ using a unit filler to allow for the accurate density determination of very small powder samples. Density measurements were performed on 0.1–0.5 g of powder. Prior to measurement, the sample chamber was purged with helium for 20 cleaning cycles at 20°C. A total of 5 sample measurements within a maximum error of 1% was used to calculate the reported density. Tap density (ρ tap) was determined according to Method A in document QAS/11.450, published by the WHO in 2012 for addition to the International Pharmacopeia. However, instead of a 250mL volumetric glass cylinder, a converted volumetric 5mL pipette tube was used to measure the small powder samples. The machine used for tapped density determination was built in-house by the IBME workshop. Briefly, a small electronic motor moves a circular brass disc according to a pre-set speed and raises a metal plateau upon which the graded volumetric plastic pipette cylinder is attached. The plateau with the sample containing cylinder is raised about 1cm in height and drops by force of gravity alone, before being raised and dropped again. While the tap height remains constant, the tap frequency can be adjusted based on the supplied voltage to the motor, which alters the rotational speed of the disc. The total number of taps required according to the International Pharmacopeia recommendation before each measurement was divided by the frequency (taps per minute). The thus calculated required time (min) was kept using a stopwatch. From helium pycnometry and tapped density values, the porosity of particles was calculated according to the equation:

$$\varepsilon =1-\frac{\rho_{tap}}{\rho_{He}}$$ (1)

Powder flowability was calculated using Carr’s compressibility index, which relates the free powder bulk density to the tapped bulk density according to the equation:

$$C=100(1-\frac{\rho_{bulk}}{\rho_{tap}})$$ (2)

### Particle size analysis with laser light diffraction

Size volume and number distribution of particles were obtained on a Malvern Mastersizer S (Malvern Instruments Ltd, Malvern, UK), in a small volume dispersion cell. A 300RF lens with backscatter detector with an active beam length of 14.3 mm was used to obtain an average of 6 times 6000 measurements according to Mie theory. Particles were added until 10–13% detector obscuration was achieved at 2000 rpm stirring rate. Particles were characterized by median diameter, D(v, 0.5) and span of the volume distribution:

$$Span=\frac{d(v,0.9)-d(v,0.1)}{d(v,0.5)}$$ (3)

D(v,0.5) corresponds to the median diameter of the volume distribution, and d(v,0.1) and d(v,0.9) to the particle diameter at 10% and 90% of the cumulative distribution. Refractive indices for SFD particles (RI 1.5376) in isopropanol (RI 1.3776) were used in the method.

### Karl-Fisher Titration- residual moisture determination

A Karl-Fischer DL39 titrator with Stromboli oven and autosampler (Mettler Toledo, Leicester, UK) was used to determine the residual moisture content of 100 mg powder samples. Titration was performed at 125°C for 30 min. Results are reported as%wt. of water relative to the powder mass.

### Scanning electron microscopy

The morphology of powder particles was examined on an Amray 1810 T Scanning Electron Microscope (SEM) at 20 kV. The samples were fixed on an aluminium disc (model G 301, Plano) using a carbon adhesive sheet. Samples were gold-sputtered at 20 mA and 5 kV (Hummer JR Technics, Germany) for 1–5 min prior to imaging.

### SDS-PAGE

SDS-PAGE used the Hoefer SE250 (Hoefer, Holliston, MA) set-up and a PowerPac (BioRad, Hercules, CA) power unit in combination with a precast 4 – 20% Precise Tris-Glycine gel, 15-Well (Thermo Scientific, Piers Protein Research, USA) and the PrecisionPlus Protein Dual-colour 500 μl (BioRad, USA). Running buffer (5x- concentrated) was prepared from 14.5 g Tris base, 72 g glycine, 5 g SDS in 1 L MilliQ water, and filtered with 0.22 μm Millipore SteriCup. Sample stain comprised of 2 mL 1 M Tris-Cl pH6.8, 6 g glycerol, 1.6 g SDS, 4 mg Brilliant Blue, 0.62 g DTT, for a 20 mL final volume. The staining solution comprised of 0.05% (w/v) Coomassie R Brilliant Blue (CBB), 10% (v/v) acetic acid, 45% (v/v) methanol, 45% (v/v) milliQ water, and the destaining solution of 10% (v/v) acetic acid, 30% (v/v) methanol, 60% (v/v) milliQ water. Sample concentrations ranged between 20 μg/mL to 300 μg/mL, and 20 μL were used per well, and 5μL of the PrecisionPlus protein ladder (BioRad, UK). Gels were run for 45 min at 185 V. After the run, the SDS-PAGE gel was rinsed in milliQ water for 5min with 3 complete changes. The gel was stained overnight in a sealed tupperware-box placed on a rocker. Destaining was conducted for 4hours or until the gel was clear, with at least 2 full changes of destaining solution. The gel was imaged on Gel DocTM XR (BioRad, USA) with Quanti One 4.66 software and analysed in GelAnalyser2010 (Freeware, http://gelanalyzer.com/).

### Immunisation experiments in mice

Procedures were performed according to the U.K. Animals (Scientific Procedures) Act 1986 and were approved by the University of Oxford Animal Care and Ethical Review Committee. Six to 8-week-old female BALB/c-OlaHsd mice (Harlan, UK) were housed in specific pathogen-free conditions. The vaccine dose per mouse was 8 μg of CRM with 5μg of MenY polysaccharide with or without 85μg of Alhydrogel® (Alum) (Benntag Biosector, Denmark) as stated in figure legends. The ratio of polysaccharide to protein in the MenY-CRM_197_ vaccine was 0.625. The vaccines were given either intramuscularly (IM) to both hind thigh muscles, intradermally (ID) to the pinna of a single ear or by needle free powder injection to the pinna of a single ear. There was a four-week interval between priming and boosting immunizations. Blood from tail bleeds or terminal cardiac bleeds was collected into Eppendorf tubes at day 28 and day 42 after the priming immunization. Blood was allowed to clot and centrifuged at 15,000 x g for 10 minutes. Sera were aliquoted and stored at ‒20°C until use.

### Meningococcal serotype Y polysaccharide ELISA

Meningococcal capsular group Y polysaccharide-specific IgG antibodies were measured by ELISA following a previously described method [40]. Briefly, immulon-2 microtiter plates (Thermo Fischer Scientific, MA, USA) were coated with capsular group Y meningococcal polysaccharide (5 μg/ml) (NIBSC, England) conjugated to 5 μg/ml of methylated human albumin (NIBSC, England) in sterile PBS. Following blocking, two-fold dilutions of test sera were assayed in duplicate. After overnight incubation at 4°C, microtiter plates were developed with HRP-conjugated goat anti-mouse secondary antibody (Jackson ImmunoResearch Inc. PA, USA) at a 1:10,000 dilution for 2.5 h at room temperature, followed by the chromogenic substrate tetramethylbenzidine (Sigma–Aldrich, England). The reaction was stopped after 30 min with 2 M H_2_SO_4_. The optical density of each well was read at 450 nm. Endpoint-titres were determined as the reciprocal of the dilution giving an OD450 nm reading above that obtained for naive control wells plus 2 x SD of 6 replicates for each plate.

### Serum bactericidal assay (SBA)

Bactericidal antibody responses were measured using SBA with human complement, as previously described [41]. The SBA was performed on serum from individual mice two weeks after the second dose of MenY-CRM_197_. Exogenous human complement (serum) without intrinsic bactericidal activity was sourced from a consenting healthy adult and used at 25% (vol/vol). Mouse sera were heat inactivated at 56°C for 30 minutes to remove intrinsic complement. The capsule group Y meningococcal strain 860800 Y:P5-1,10–4: F4-1:ST-29 (cc167) was grown overnight on blood agar plates at 37°C with 5% CO_2_. Approximately 50 colonies were sub-cultured for 4 hours, and reconstituted in Hanks buffered salt solution (Thermo Fischer Scientific, MA, USA) with 0.5% bovine serum albumin (Sigma Aldrich, MO, USA). The bacteria were diluted to give approximately 100 colony forming units per 10μl used for the assay. The SBA titre was defined as the reciprocal of the highest dilution of serum that yielded ≥50% decrease in CFU relative to that of control wells within 60min at 37°C.

## Results

### Addition of Polysorbate 80 to Trehalose-Mannitol-Dextran10kDa-Dextran150kDa (TMDD) does not reduce aggregation of MenY-CRM_197_ during SFD relative to TMDD alone

**Fig 2:**
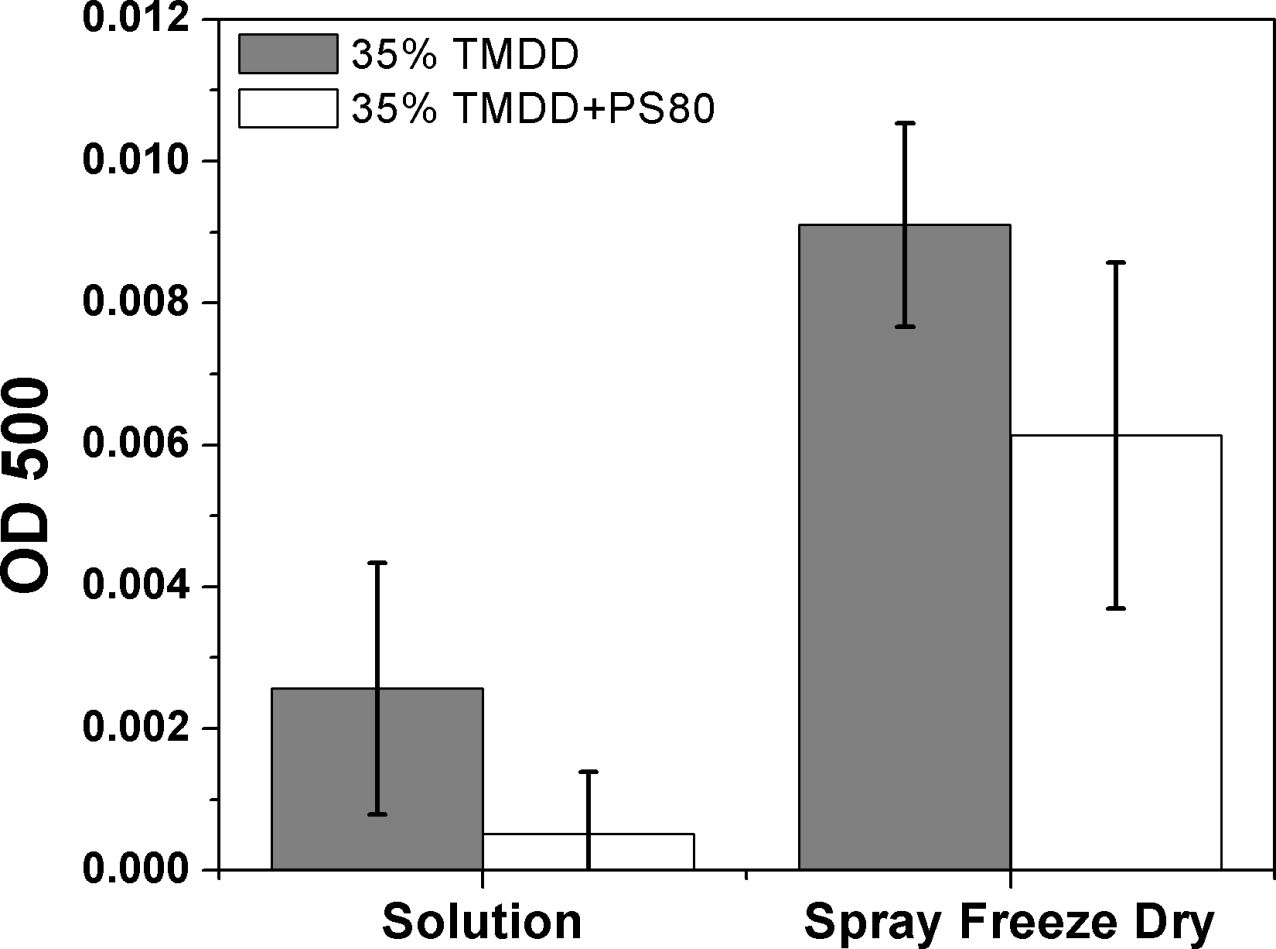
**Turbidity measurements (OD**_**500**_**) of 2 mg/mL MenY-CRM**_**197**_ **a) in solution, and after b) atomisation, freezing and lyophilisation and resuspension.** Solutions contained 35% wt (3:3:3:1) trehalose, mannitol, dextran (10kDa), dextran (150kDa), and 35% wt. TMDD with 0.1% (w/w) Polysorbate 80, in 10mM K_2_HPO_4_ buffer pH 7.2. The addition of PS80 to TMMD did not result in significantly lower turbidity (OD_500_) of the resuspended powder vaccine.

Protein aggregates may induce cytotoxicity and misguided antibodies [42]. Therefore, we tested TMDD formulations for visible aggregates of CRM_197_ before and after spray-freeze-drying (SFD) using light scattering (λ = 500 nm). We investigated whether the addition of the surfactants Polysorbate 80, Polysorbate 20, Poloxamer K188, or Kolliphor HS, at three different concentrations 0.01% wt. 0.1%wt, and 1% wt reduces protein aggregation relative to the standard TMDD (3:3:3:1) matrix. The high surfactant concentration (1% wt.) was excluded from further formulations due to visible foaming during the spray process. Of the tested surfactants at 0.01 and 0.1 wt.%, Polysorbate 80 showed the least CRM_197_ loss during SFD (data not shown). Polysorbate 80 (0.1% wt.) does not reduce protein aggregation during SFD relative to the standard TMDD (3:3:3:1) matrix (Fig 2). MenY-CRM_197_ liquid formulations with 35% (w/w) TMDD (2.6 ± 1.8 mOD_500_) and 35% (w/w) TMDD-PS80 (0.5 ± 0.9 mOD_500_) showed low OD_500_ levels and were not different from one another before and after SFD. After ultrasound atomisation, OD_500_ levels increased approximately 3.5-fold for TMDD and 12-fold for TMDD+PS80, and were significantly higher than OD_500_ levels of the initial liquid TMDD (9.1 ± 1.4 mOD_500_) and 35% TMDD-PS80 (6.1 ± 2.4 mOD_500_) formulations. BCA assay indicated a 4% loss of protein after SFD (4.7 ± 0.2 μg/mL) relative to stock solutions (4.9 ± 0.1 μg/mL) for both formulations. To keep the formulation and subsequent analysis simple, 35% (w/w) TMDD (3:3:3:1) without PS80 was used for dry-powder vaccine formulation.

### SFD dry-powder formulation creates vaccine-loaded microparticles with sufficient physical robustness for needle-free powder injection

**Fig 3:**
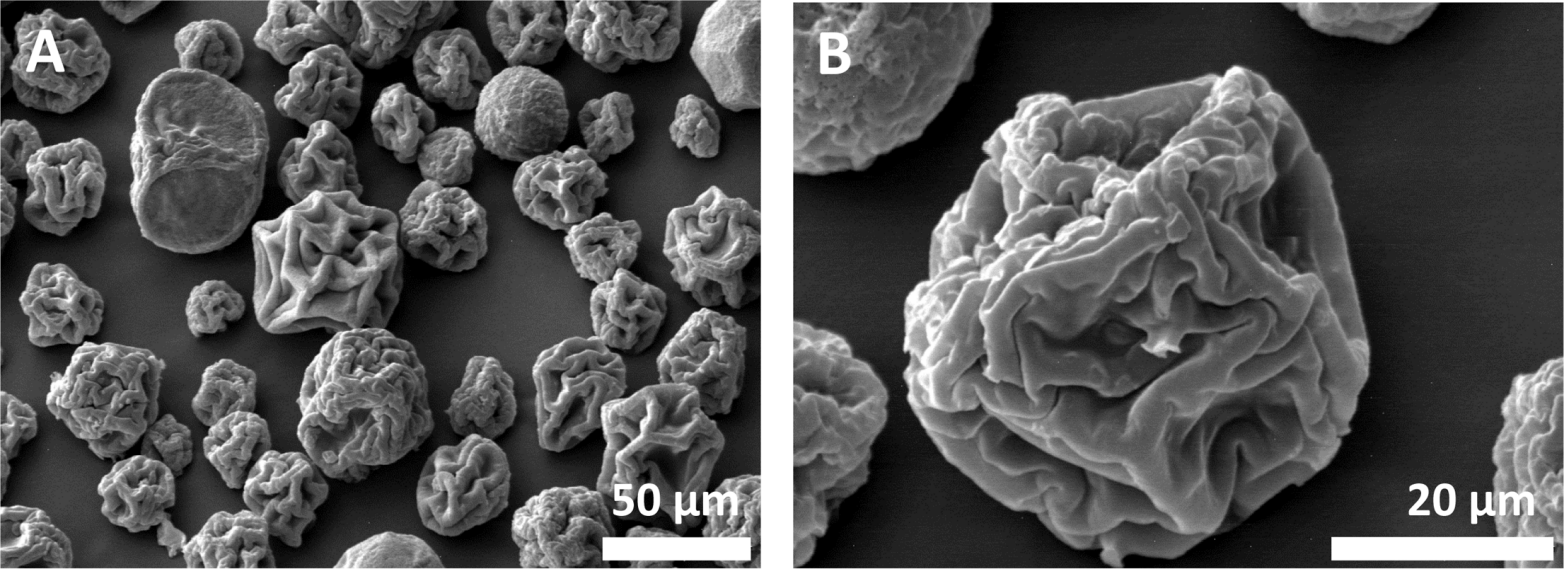
**Microparticle size distribution (A) and surface morphology (B) by SEM.** Microparticles with average diameter of 58.1 μm were produced by SFD from a 35% (w/w) TMDD-MenY-CRM_197_ (3:3:3:1) solution, lyophilized at -20°C and 100 mTorr (0.13 mbar) for 72 hours (primary drying) and at 20°C and 100 mTorr for 24 hour (secondary drying,) show a wrinkled surface morphology characteristic of trehalose-manitol-dextran particles.

To enable pain-free intradermal delivery, vaccine particles need to breech the *stratum corneum*, which requires a particle impact parameter of 7 – 12 kg/m·s, a particle size less than 100 μm, and sufficient physical robustness. Lyophilisation of the frozen TMDD droplets above their glass transition temperature (T_g_’ = ‒29.2 ± 0.4°C) produced free-flowing powders that comprised of micron-sized individual particles with the characteristic wrinkled surface morphology (Fig 3). Particles contain 0.5% wt. MenY-CRM_197_ (mass of MenY-CRM_197_ to mass of excipients), unchanged from the pre-SFD suspension.

TMDD and MenY-CRM_197_-TMDD particles had mean diameters of 57.4 μm and 58.1 μm respectively (Table 1). The bulk density of the TMDD particles corresponded to literature values of the sugars, approximately 1.60 g/cm^3^ ± 1% (Table 1). The tap density of particles was 3.4-fold lower (0.47 ± 0.07 g/cm^3^), which translates to an average particle porosity of 70.6%, with a high glass transition temperature (T_g_ = 42.8 ± 0.5°C) and low residual moisture content 0.6 ± 0.2% (w/w) of the final SFD powder formulation.

**Table 1:** Physical characteristics of matrix and vaccine SFD formulations.

Formulation property	TMDD	TMDD-MenY-CRM_197_
Solid Content (% wt.)	35	35
TMDD Relative Ratio	3:3:3:1	3:3:3:1
MenY-CRM content (% wt.)	0.0	0.5
1° Drying (°C, mTorr, hrs)	-20, 100, 72	-20, 100, 72
2° Drying (°C, mTorr, hrs)	20, 100, 24	20, 100, 24
Yield (% wt. solutes/powder)	92 ± 7%	90 ± 8%
Residual H_2_O (% wt.)	0.6 ± 0.2	3.5 ± 1.5
Tg' (°C)	-29.2 ± 0.4	-29.9 ± 0.3
Tg (°C)	42.8 ± 0.5	45.2 ± 0.5
dCp (J/g°C)	0.69 ± 0.01	0.45 ± 0.03
D(v, 0.5) (μm), (Span)	57.4 (1.3)	58.1 (1.2)
He-Pycnometry (g/cm^3^)	1.60 ± 1%	1.49 ± 1%
Tapped Density (g/cm^3^)	0.47 ± 0.07	0.53 ± 0.09
Carr’s Index	11.3 ± 0.8	15.7 ± 1.4
Porosity (%)	70.6	64.4
Impact Parameter (ρvr)	10.1	11.5

The addition of 0.5% (w/w) MenY-CRM_197_ to the 35% (w/w) TMDD (3:3:3:1) matrix changed several physical properties of the particles. The particles were less porous (64.4%) than pure TMDD particles, with a bulk density of 1.49 g/cm^3^ ± 1% and a 2.8-fold lower tap density (0.53 ± 0.09 g/cm^3^). A higher glass transition temperature (T_g_ = 45.2 ± 0.5°C) and a higher residual moisture content 3.5 ± 1.5% (w/w) were observed. The glass transition temperature (T_g_’ = -29.2 ± 0.4°C) was not different from the pure TMDD freeze-concentrate, and therefore the same lyophilisation program was used.

The observed particle diameters and the calculated impact factors for TMDD (10.1 kg/m·s) and TMDD-CRM_197_ (11.5 kg/m·s) fall within the recommended range for pain-free intradermal powder injection [16, 20].

### Chemico-physical integrity of MenY-CRM_197_ is maintained during SFD

**Fig 4:**
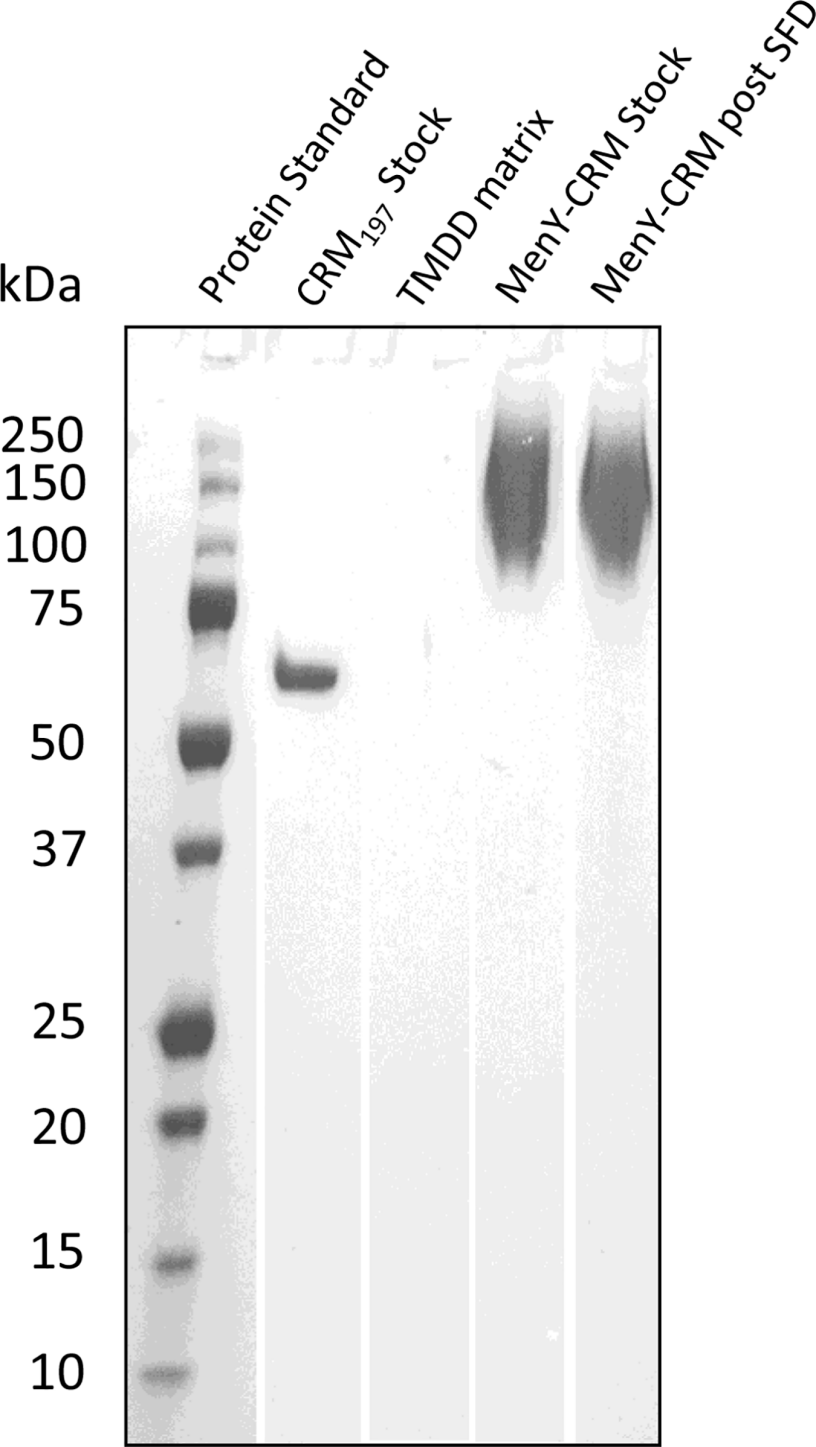
Chemico-physical integrity of MenY-CRM_197_ assessment by SDS-PAGE. SDS-PAGE used precast 4–20% Precise Tris-Glycine gels and the PrecisionPlus Protein Dual-colour protein standard. Samples were applied to the 15-well gel in every other lane. Control lanes were loaded with CRM_197_ stock protein (2.0 ng), 35% (w/w) TMDD matrix without MenY-CRM_197_ (10 μg), untreated MenY-CRM_197_ stock (12 ng), and resuspended SFD MenY-CRM_197_ (12 ng) SFD powder.

Aggregation of biotherapeutic proteins has been linked to cytotoxicity, decreased biological activity, and increased immunogenicity. According to SDS-PAGE, the 58.4 kDa [43] CRM_197_ protein control showed a defined band at 60 kDa (Fig 4). The untreated control MenY-CRM_197_ and resuspended dry-powder MenY-CRM_197_-TMDD bands appear equivalent to each other, without detectable hydrolysis products in the lower MW band region. Detectable fractions ranged from 300 kDa to 75 kDa (Table 2). The wide range of MenY-CRM_197_ molecular weights (band smear) in the stock and post-SFD lanes arises from heterogeneous MenY polysaccharide substitution to the CRM_197_ carrier protein [44, 45]. Asymmetric flow field-flow fractionation of the MenY-CRM_197_ vaccine before and after SFD showed a 93% ± 6% recovery of MenY-CRM_197,_ and did not detect physical aggregates (see Figure A S1 File). The total protein mass detected in the band intensities correspond to the nominal values (12ng MenY-CRM_197_), as calculated by the integration of the intensity bands relative to a CRM_197_ standard curve.

**Table 2:** Semi-quantitative size analysis of MenY-CRM_197_ by SDS-PAGE.

SDS-PAGE analysis	CRM_197_ Stock	MenY-CRM_197_ Stock	MenY-CRM_197_ Post SFD
Total mass (μg) in lane (vs. nominal)	2.2 (2.0)	11.9 (12.0)	11.6 (12.0)
Upper bound (kDa)	66	1790	1590
Median (kDa)	62	150	149
Lower bound (kDa)	58	77	74
Aggregates (%)	ND	ND	ND
Hydrolysis products	ND	ND	ND

### Delivery of MenY-CRM_197_ vaccine by powder injection induces MenY-polysaccharide specific antibodies

**Fig 5:**
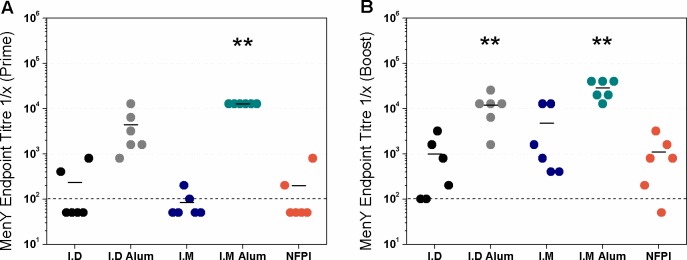
**MenY polysaccharide-specific IgG responses to MenY-CRM**_**197**_ **prime (A) and boost (B) administered IM, ID or by needle free powder injection.** Total IgG end-point titres in Balb/c mice were measured by MenY-polysaccharide ELISA after two 5μg doses of MenY-CRM_197_ delivered either ID, IM or by needle free powder injection on days 28 and 42. ID and IM vaccinations were performed with or without Alum as indicated on the X axis. The geometric mean values for each group are indicated by bars. The limit of detection is indicated by the dashed line. n = 6 per group. ** = P < 0.01 by Kruskal-Wallis with Dunns post-test compared with pooled sera of naïve mice. Values below 100 were designated an arbitrary value of 50.

Having established that the SFD process could produce particulates with the desired characteristics without impacting on the immunogen structure, we sought to compare the humoral response in Balb/C mice after administration of a MenY-CRM_197_ vaccine using a needle free powder injector (NFPI), to intradermal (ID) or intramuscular (IM) delivery. Two doses of MenY-CRM_197_ were given to groups of mice with a four-week interval. The ID and IM vaccines were formulated with or without Alhydrogel (Alum) although the MenACWY-CRM vaccine (Menveo, GSK) is not adjuvanted but the use of Alum in our experiments would provide a more rigorous comparison. ELISAs were performed to determine the titres of MenY-polysaccharide specific IgG antibodies for each of the different groups after two doses of the vaccine (Fig 5B). All groups had measurable antibody responses but one mouse out of 6 in the MenY-CRM_197_ NFPI group had an antibody level below the limit of detection (equivalent to naïve mice). Mice in the MenY-CRM_197_ IM + Alum group had the greatest geometric mean antibody titre (26257, 95% CI 15714, 43872), which was significantly greater (P < 0.01) than that of the MenY-CRM_197_ ID group (449, 95% CI 94, 2125) and the MenY-CRM_197_ NFPI group (566, 95% CI 117, 2739). All other comparisons between the MenY-CRM_197_ IM and MenY-CRM_197_ ID + Alum groups (1796, 95% CI 332.9, 9688 and 9051, 95% CI 3320, 24673 respectively) were not significant (P > 0.05).

Pooled sera from mice in the MenY-CRM_197_ IM + Alum and the MenY-CRM_197_ NFPI groups were used to perform IgG1, IgG2a, IgG2b and IgG3 subclass ELISAs against MenY polysaccharide. This was to investigate whether there was a difference in the subclasses induced by the different routes of vaccine administration. The responses were very weak or undetectable for all of the subclasses (data not shown) with the exception of IgG1 where the OD450nm reading was 2.8 for the MenY-CRM_197_ IM + Alum group compared with OD450nm = 0.5 for the MenY-CRM_197_ NFPI group, both at a serum dilution of 1/250 (data not shown).

### Vaccination by needle free powder injector results in comparable serum bactericidal antibody titres with ID and IM MenY-CRM197 vaccine delivery

**Fig 6:**
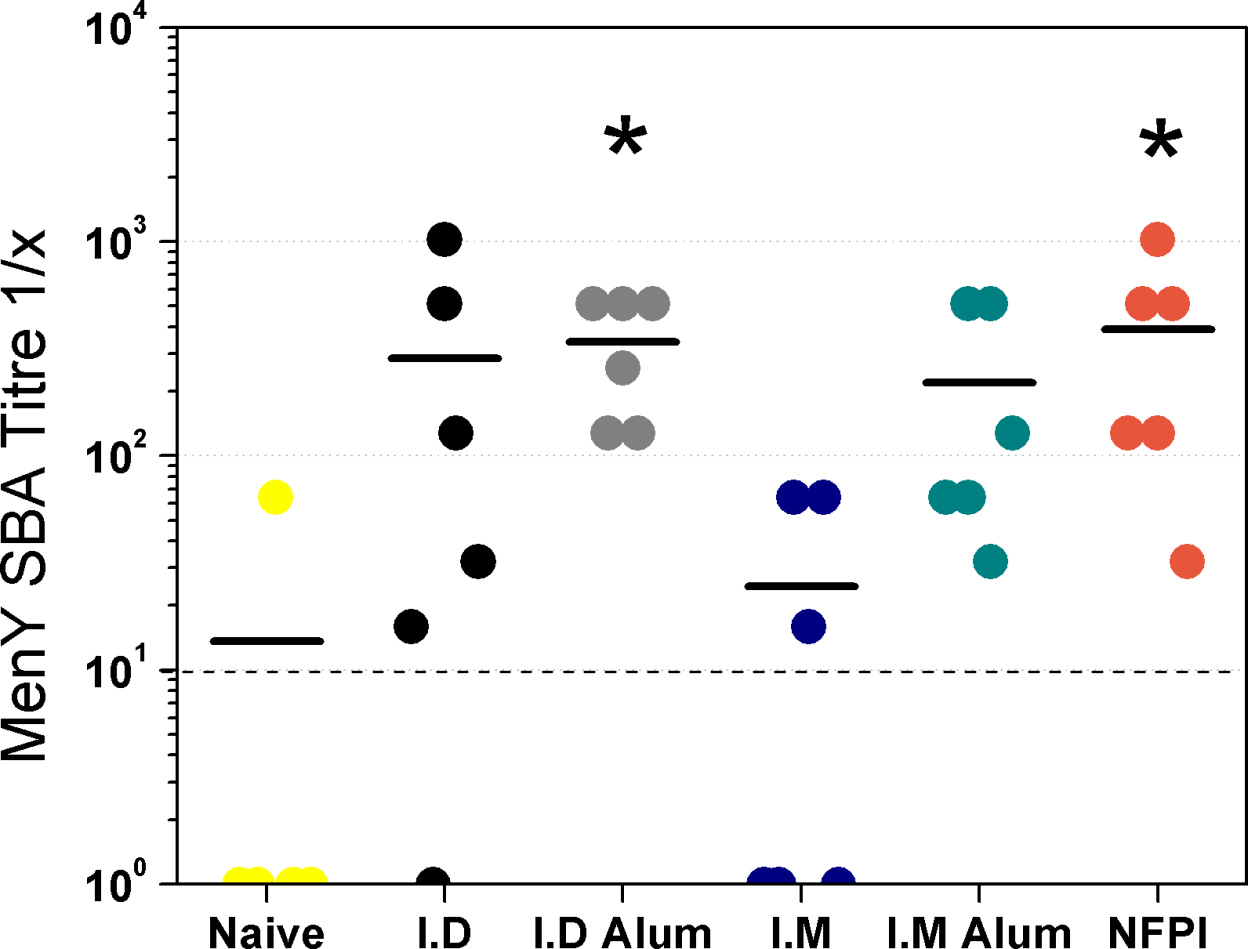
Serum bactericidal antibody responses to MenY-CRM_197_ administered IM, ID or by needle free powder injector. hSBA end-point titres in Balb/c mice were measured after two 5μg doses of MenY-CRM_197_ delivered either ID, IM or by needle free powder injector (NDPI). ID and IM vaccinations were performed with or without Alum indicated in the axis. The geometric means for each group are indicated by bars. The limit of detection is indicated by the dashed line. n = five or 6 per group. * = P < 0.05 by Kruskal-Wallis with Dunns post-test compared with Naïve group. SBA titres below 1:4 were designated an arbitrary value of 1.

Due to low incidence rates of meningococcal disease globally, efficacy trials of new vaccines are unfeasible. Therefore, meningococcal vaccines are licensed based upon a surrogate of protection, which is a serum bactericidal antibody (SBA) titre ≥1:8 when using rabbit complement (rSBA) or ≥1:4 when using exogenous human complement (hSBA). We therefore measured the bactericidal antibody responses after ID, IM or needle free powder injection administration of the MenY-CRM_197_ vaccine in the same experimental groups of mice used to perform the ELISAs (Fig 6). After two doses of vaccine the geometric mean hSBA titres in the MenY-CRM_197_ ID and MenY-CRM_197_ IM groups (both without Alum) (57.0, 95%CI 4.0, 815.0 and 6.4, 95% CI 0.7, 56.8 respectively) were not significantly greater than that of the naïve group (2.3, 95% CI 0.2, 23.1), which contained an outlier hSBA titre of 1:64 in one animal. The MenY-CRM_197_ NFPI and MenY-CRM_197_ ID + Alum groups had significantly (P <0.05) greater geometric mean hSBA titres (228.1, 95% CI 60.0, 866.6 and 287.4, 95% CI 140.5, 587.6 respectively) compared with the naïve group. None of the groups that received the vaccine by a traditional injection route had a greater mean hSBA titre than the MenY-CRM_197_ NFPI group.

## Discussion

In this study we have shown that delivery of the MenY-CRM_197_ vaccine by needle free powder injection results in bactericidal antibody responses that are comparable with alum-adjuvanted ID or IM vaccinations in mice. We have also shown that spray-freeze drying a high solute content TMDD-MenY-CRM_197_ solution produces vaccine-loaded microparticles with suitable physical characteristics for NFPI that adequately preserve MenY-CRM_197_ structure_._

To reduce the effect of the shear stress and possible hydrophobic patch association of CRM_197_ molecules at interfaces created during atomisation, Polysorbate80, a pharmaceutical grade surfactant was used. Relative to TMDD alone, the addition of surfactant showed no significant reduction in MenY-CRM_197_ loss during SFD. This is surprising in that surfactants have been shown to improve the viability of proteins during spray drying and spray-freeze drying [46, 47]. A frequently referenced mechanism of protein preservation by surfactants is their preferential accumulation at solution interfaces which can prevent protein molecules from accumulating and interacting at these interfaces [48]. These findings therefore suggest that intermolecular interactions at interfaces are not the only factor in MenY-CRM_197_ aggregation during atomisation. In addition to shear at the nozzle tip and the subsequent solution-air interface of the droplets, localised heating and high pressures due to cavitation at the ultrasound nozzle tip may lead to protein aggregation. The choice of excipients will need to balance the physico-chemical integrity of MenY-CRM_197_ with the physical requirements for the NFPI microparticles.

While sucrose and trehalose are excellent glass formers and lyo- and cryoprotectants that could preserve MenY-CRM_197_ in a dry matrix, the addition of a plasticiser is necessary to achieve the minimum mechanical robustness required for ballistic injection. Mannitol has been shown to induce substantial viscous flow compaction during freeze-drying [15, 27, 31]. The viscosity of an amorphous matrix decreases significantly at temperatures that exceed its Tg’. This behaviour is described by the Williams-Landel-Ferry equation and is a function of the difference between the product temperature (T_prod_) and Tg ‘ [49]. Plastic flow occurs usually between the Tg’ of a matrix and its collapse temperature (Tc). The collapse of an amorphous freeze concentrate occurs usually at a temperature (Tc) 2°C above its Tg’. Tang and Pikal report that plastic flow may occur when T_prod_ exceeds Tg’ or even Tc during primary drying [50]. The T_prod_ of TMDD-CRM_197_ and TMDD exceeded Tg’ during 1° drying, and the substantially reduced viscosity can cause matrix deformations by plastic flow and collapse [51]. SEM images show a wrinkled and collapsed morphology. Therefore, TMDD with and without MenY-CRM_197_, are likely compacted via plastic flow during 1°drying, which was conducted above the Tg’. Structural collapse in conventional lyophilisates would see them eliminated from further use, but in case of ballistic delivery, collapse is desirable since it increases the impact parameter. While all examined dextran-containing formulations resulted in powders physically suitable for NFPI after SFD, their ability to preserve MenY-CRM_197_ needed to be investigated.

The molecular weight distribution of a glycoconjugate is a reasonable proxy to inform vaccine immunogenicity. A decrease in immunogenicity was observed only for high degrees of physicochemical degradation of the MenC-CRM_197_, above a 30% loss of the conjugated polysaccharide, and a CRM_197_ aggregate content of 23% [52]. On average, MenY-CRM_197_ has 5 – 10 MenY polysaccharide chains per CRM_197_ monomer (58.4 kDa), each with an average degree of polymerization of 20 – 21, and an average formulation weight of 145 kDa, suggesting an average aggregate MenY content of 87 kDa per glycoconjugate molecule [43]. The presented SDS-PAGE data are in good agreement with published values and range from 75 kDa to 300 kDa, with a mean of 150 kDa. The MenY-CRM_197_ intensity bands by SDS-PAGE and AF4 elution profile suggest a similar MW distribution before and after SFD. Furthermore, the absence of lower MW bands indicates that the chemical degradation of the CRM_197_ protein after SFD was less than 1%, which is based on the assay’s 0.1 μg/band detection limit. Therefore, the chemical integrity of MenY-CRM_197_ was largely preserved during SFD.

Considering the ELISA data, there was no significant effect of the adjuvant Alhydrogel on the antibody end-point titres when the vaccine was delivered either ID or IM. This is in agreement with the finding that in infant studies, decreased immunogenicity in response to the quadrivalent MenACWY-CRM was not observed when aluminium phosphate adjuvant was omitted from the vaccine [53, 54]. However, the ELISA data indicated that mice in the MenY-CRM_197_ IM + Alum group developed the greatest amount of antibodies in response to the vaccine. By comparison, mice in the MenY-CRM_197_ NFPI group appeared to have weaker antibody responses.

The ELISA data are in stark contrast to the results seen in the hSBA. Only groups of mice that received the vaccine by needle-free powder injection or intradermally with Alum developed bactericidal antibody titres that were significantly higher than the naïve group. The discordance between the IgG titres measured by ELISA and the bactericidal antibody titres measured by hSBA are not wholly unexpected. A number of studies have shown that antibodies measured by standard polysaccharide ELISAs do not correlate well with SBA titres [55–58]. This effect has been attribute d to differences in the avidity of antibodies, since high avidity antibodies are necessary to induce complement mediated bacterial lysis in the SBA. It has been shown that avidity ELISAs have a stronger correlation with SBA compared with standard ELISA [57]. It is reasonable therefore, that the needle-free powder injector induced fewer antibodies than IM delivery with Alum, but those antibodies were of higher avidity, and resultantly, were more bactericidal.

The different routes of vaccine administration could have resulted in different IgG subclasses to be induced. In mice, IgG1 is known to be the main bactericidal subclass produced in response to protein-conjugated polysaccharide vaccines [59]. The only subclass detected was IgG1 which was present in the serum from the MenY-CRM_197_ IM + Alum and the MenY-CRM_197_ NFPI groups. Therefore, it does not appear that the bactericidal antibodies induced by needle-free powder injection were of a different subclass than those induced by IM vaccination. Mice that received the vaccine by ID injection to the ear had similar humoral responses to those that received the vaccine by the powder injector. There is evidence that ID vaccination generally results in stronger immune responses compared with IM vaccination [60–62]. The most likely explanation for this is that the dermis contains a greater proportion of antigen presenting cells such as Langerhans cells, which take up antigens and migrate to lymph nodes where they present their bound antigen to T and B cells [24]. This study shows that needle free powder injection of the MenY-CRM_197_ conjugate vaccine results in bactericidal antibody responses in mice that are equivalent to ID and IM delivery.

The struggle to prevent disease globally is perpetual. While much progress has been made, vaccines and delivery strategies can be improved further. Needle-free intradermal immunisation may expand the medical arsenal that is used to combat infectious diseases. Our results with MenY-CRM_197_ demonstrate that needle free vaccination is both technically and immunologically valid, and could be considered for a broader range of glycoconjugate vaccines in development.

## Supporting information

S1 File AF4 analysis at OD 280nm compared the controls CRM_197_ (10ug), MenY-CRM_197_ stock solution (10ug), versus the resuspended SFD powders containing MenY-CRM_197_. (DOCX)
