# Supplementary material for: Alternative vaccine administration by powder injection: Needle-free dermal delivery of the glycoconjugate meningococcal group Y vaccine
Source: PLoS One. 2017 Aug 24;12(8):e0183427. doi: 10.1371/journal.pone.0183427 (PMC5570268; doi:10.1371/journal.pone.0183427)
Supplement: S1 File — (DOCX) [file pone.0183427.s001.docx]

**AF4 analysis at OD 280nm compared the controls CRM_197_ (10ug), MenY-CRM197 stock solution (10ug), versus the resuspended SFD powders containing MenY-CRM_197_.**

**Materials and methods**

MenY-CRM_197_ samples were suspended in 0.1μm-filtered PBS pH7.4 running buffer before injection into the AF4 (AF2000MT, PostNova Analytics, Landsberg, GER). Measurements were conducted at room temperature. Protein concentrations were determined with a UV-Vis unit from Shimadzu (SPD-20A, Postnova, PN3211) at wavelength λ=280nm. The fractionation chamber was equipped with a 10kDa MWCO regenerated cellulose (RC) membrane (Postnova, Z-AF4-MEM-612) and a 500μm spacer. Prior to sample analysis, the system was flushed with running buffer with the flow rates of 2mL/min, 2mL/min, 3mL/min for tip, focus and cross flow respectively for about 15min. The 20 μL sample was injected into the column with a flow rate of 0.2mL/min. The cross flow during the sample focussing step was set to 4mL/min for 5min. The detector flow rate was 0.4mL/min and the slot flow rate 0.6mL/min. After 20min at 4mL/min cross flow, a linear gradient of the cross flow from 4mL/min to 0.0mL/min over 5min. To ensure that any possible aggregates eluted from the fractionation chamber, a constant flow was maintained in the chamber with 1mL/min tip flow and 0.4mL/min detector flow, and 0.6mL/min slot flow for 25min. The UV signal of a given elution profile were baselined in the AF2000 software version 1.1 (PostNova Analytics, Landsberg, GER). Regions of interest were defined for each elution fraction. The viscosity of the elution buffer was 0.0088g/cms and had a refractive index of 1.330. The MenY-CRM_197_ sample concentration was used at 1mg/mL. The extinction coefficient of CRM_197_ was previously determined to be εCRM=0.759 [mL/(mg*cm)]. SFD powders with 2% wt MenY-CRM197 were resuspended in 10mM K2HPO4 pH7.2 at 10mg in 0.5mL. The resuspended solution was concentrated in centrifuge tubes (Corning Spin-X UF, 10k MWCO) at 2,000xg for 5min, and resuspended to 0.25mL with K_2_HPO_4_ buffer for a final CRM_197_ concentration of 1mg/mL.

**SI - Results**

Comparison of the MenY-CRM vaccine before and after SFD by asymmetrical flow field-flow fractionation showed a 93% ± 6% recovery of MenY-CRM_197_ for the SFD process as determined by the absorbance intensity integration of the baselined elution diagram at wavelength 280nm (S1 Fig. A). The resuspended MenY-CRM_197_ vaccine showed an 11% ± 4% decreased AUC relative to the stock vaccine between t=0-24min, and a 8% ± 5% increased AUC over the stock vaccine for t=24-60min. An increase in high molecular weight aggregates was not detected.

**SI -** **Discussion**

On average, the MenY-CRM_197_ has 5-10 MenY polysaccharide chain conjugations per CRM_197_ carrier protein [1]. The broad elution profile of the MenY-CRM_197_ stock vaccine, relative to the defined CRM_197_ monomer elution peak suggests a heterogeneous substitution of the MenY polysaccharide to the CRM_197_ carrier. At a lower MenY substitution density, more protein surface is exposed which may lead to protein-protein interaction and association. The observed shift in AUC of comparable amount from the lower molecular weight fractions to higher molecular weight fractions with the MenY-CRM_197_ elution profile suggests that minimally glycosylated MenY-CRM_197_ molecules may physically associate during SFD. At higher MenY substitutions, steric hindrance between the MenY polysaccharides makes protein-protein interactions less frequent. The absence of high molecular weight aggregates (>50min) suggests that highly glycosylated MenY-CRM_197_ molecules are unlikely to associate during SFD. The impact of the SFD process on MenY-CRM_197_ does not form oligomers that are larger than the molecular sizes contained in the stock vaccine (S. Fig A). The absence of visible (≥10μm) or sub-visible (2-10μm) particles makes the formulation compliant with US Pharmacopeia guidance [2, 3].

**SI - References:**

1. Berti F, Costantino P, Fragai M, Luchinat C. Water accessibility, aggregation, and motional features of polysaccharide-protein conjugate vaccines. Biophys J. 2004;86(1 Pt 1):3-9. doi: 10.1016/S0006-3495(04)74078-3. PubMed PMID: 14695244; PubMed Central PMCID: PMC1303793.

2. Das TK. Protein particulate detection issues in biotherapeutics development—Current status. AAPS PharmSciTech. 2012;13(2):732-46.

3. <788>; U. Particulate matter in injections. MD, USA: The United States Pharmacopeial Convention. 2012;(USP 35–NF 30).


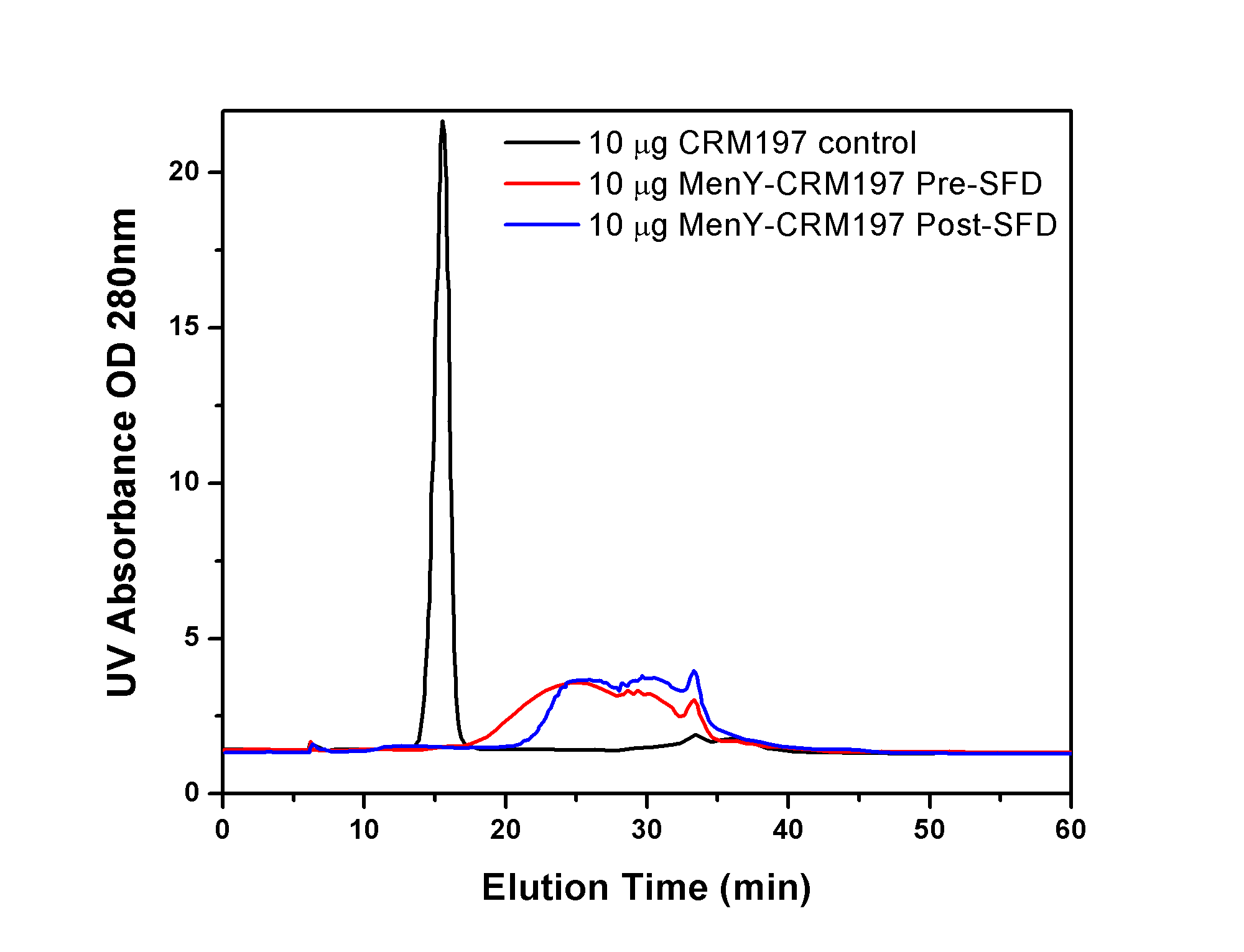


**Figure A. AF4 analysis at OD 280nm compared the controls CRM_197_ (10ug), MenY-CRM_197_ stock solution (10ug), versus the resuspended SFD powders containing MenY-CRM_197_**. Running buffer was 0.1μm-filtered PBS pH7.4, and flow rates were 1mL/min tip flow 0.4mL/min detector flow, and 0.6mL/min slot flow. To separate the injected sampled (20uL) by molecular mass, a 4mL/min cross flow was applied for 4min followed by a linear gradient from 4mL/min to 0mL/min over 5min.
